# Defining gene ends: RNA polymerase II CTD threonine 4 phosphorylation marks transcription termination regions genome-wide

**DOI:** 10.1093/nar/gkae1240

**Published:** 2024-12-24

**Authors:** Magda Kopczyńska, Upasana Saha, Anastasiia Romanenko, Takayuki Nojima, Michał R Gdula, Kinga Kamieniarz-Gdula

**Affiliations:** Center for Advanced Technologies, Adam Mickiewicz University, Uniwersytetu Poznanskiego 10, 61-614 Poznan, Poland; Department of Molecular and Cellular Biology, Institute of Molecular Biology and Biotechnology, Faculty of Biology, Adam Mickiewicz University, Uniwersytetu Poznanskiego 6, 61-614 Poznan, Poland; Center for Advanced Technologies, Adam Mickiewicz University, Uniwersytetu Poznanskiego 10, 61-614 Poznan, Poland; Department of Molecular and Cellular Biology, Institute of Molecular Biology and Biotechnology, Faculty of Biology, Adam Mickiewicz University, Uniwersytetu Poznanskiego 6, 61-614 Poznan, Poland; Center for Advanced Technologies, Adam Mickiewicz University, Uniwersytetu Poznanskiego 10, 61-614 Poznan, Poland; Medical institute of Bioregulation, Kyushu University, 3-1-1 Maidashi, Higashi-ku, Fukuoka 812-8582, Japan; Center for Advanced Technologies, Adam Mickiewicz University, Uniwersytetu Poznanskiego 10, 61-614 Poznan, Poland; Department of Gene Expression, Institute of Molecular Biology and Biotechnology, Faculty of Biology, Adam Mickiewicz University, Uniwersytetu Poznanskiego 6, 61-614 Poznan, Poland; Center for Advanced Technologies, Adam Mickiewicz University, Uniwersytetu Poznanskiego 10, 61-614 Poznan, Poland; Department of Molecular and Cellular Biology, Institute of Molecular Biology and Biotechnology, Faculty of Biology, Adam Mickiewicz University, Uniwersytetu Poznanskiego 6, 61-614 Poznan, Poland

## Abstract

Defining the beginning of a eukaryotic protein-coding gene is relatively simple. It corresponds to the first ribonucleotide incorporated by RNA polymerase II (Pol II) into the nascent RNA molecule. This nucleotide is protected by capping and maintained in the mature messenger RNA (mRNA). However, in higher eukaryotes, the end of mRNA is separated from the sites of transcription termination by hundreds to thousands of base pairs. Currently used genomic annotations only take account of the end of the mature transcript – the sites where pre-mRNA cleavage occurs, while the regions in which transcription terminates are unannotated. Here, we describe the evidence for a marker of transcription termination, which could be widely applicable in genomic studies. Pol II termination regions can be determined genome-wide by detecting Pol II phosphorylated on threonine 4 of its C-terminal domain (Pol II CTD-T4ph). Pol II in this state pauses before leaving the DNA template. Up to date this potent mark has been underused because the evidence for its place and role in termination is scattered across multiple publications. We summarize the observations regarding Pol II CTD-T4ph in termination regions and present bioinformatic analyses that further support Pol II CTD-T4ph as a global termination mark in animals.

## Graphical Abstract

**Graphical Abstract:**
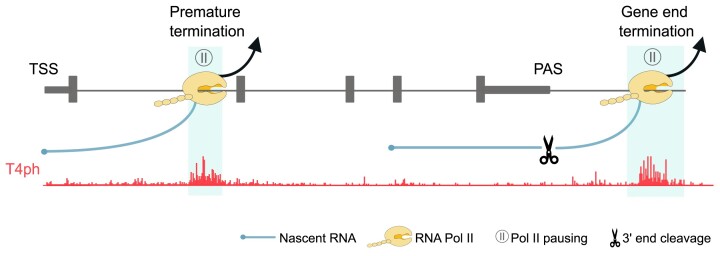

## Introduction

### RNA polymerase II-mediated transcription and its termination

Transcription by RNA polymerase II (Pol II) stands at the heart of the complex, well-orchestrated gene expression pathway in eukaryotes. Pol II is responsible for transcription of all messenger RNA (mRNA) as well as many non-coding RNA. It consists of 12 subunits, among which RPB1 is the largest and has a regulatory C-terminal domain (CTD). Navigating through initiation, elongation and termination, Pol II associates with numerous multi-subunit protein complexes. Such associations are frequently mediated by the CTD, which serves as a binding platform that allows cross-talk between Pol II-dependent RNA biogenesis pathways (1–15).

Transcription termination is a phase of the transcription cycle that is insufficiently explored. This is partially due to a common misconception that it does not matter where transcription of a gene ends. However, work from the Proudfoot lab and others, beginning in the 1980s, has repeatedly demonstrated the importance of timely termination, as delayed termination leads to transcriptional interference, typically downregulating expression of the downstream gene. (16–19). In the past decade, it has become evident that various forms of environmental stress, viral infections, several cancer types and cellular senescence are all associated with defects in transcription termination, leading to read-through transcription [reviewed in (20)]. Read-through transcription can result in RNA chimeras, aberrant oncogene expression or circular isoforms, and may even produce functionally novel proteins (21–23). On the other hand, terminating too early generates truncated gene products and is also associated with human disease [reviewed in (24)].

Mechanistically, transcription termination has two components, initially described as separate models but now stands unified. The first component involves allosteric changes in Pol II after transcribing a polyadenylation signal. The second component is a molecular ‘torpedo,’ namely a 5′-3′ exonuclease called XRN2 in mammals. XRN2 gains access to the nascent RNA following endonucleolytic cleavage at the polyadenylation site (PAS) and facilitates the displacement of Pol II from the DNA template (25,26). Very recent structural studies of archaeal and yeast pre-termination complexes confirmed that 5′-3′ exonucleolytic cleavage of RNA and concomitant 5′-3′ translocation of the exonuclease applies a mechanical force to the elongation complex and triggers termination (27,28). Both allosteric changes and successful Pol II catch up of the exonuclease torpedo are thought to be facilitated by – or even require – pausing of Pol II in termination regions (29–33).

**Figure 1.**
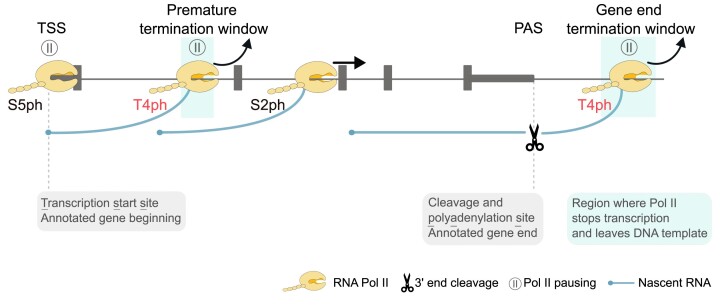
Schematic depiction of RNA Pol II transcription cycle on a metazoan protein-coding gene. Transcription initiates at the transcription start site (TSS). The heptapeptide repeats of the CTD of the largest subunit of initiating Pol II are phosphorylated on serine 5 (S5ph). Elongating Pol II is characterized by phosphorylation on serine 2 (S2ph). At an active cleavage and PAS, the nascent RNA is cleaved and polyadenylated; however, Pol II continues to transcribe further, in human cells typically thousand base pairs downstream. Pol II then undergoes terminal pausing, marked by phoshorylation of threonine 4 (T4ph), before ceasing transcription, releasing the transcript and dissociating from the DNA template. Besides PAS-linked transcription termination upon full-length mRNA production, transcription can also terminate prematurely, downstream of an intronic cryptic PAS. Also such prematurely terminating Pol II is marked by T4ph.

Besides normal termination at gene ends, protein-coding genes undergo widespread premature transcription termination, also called transcription attenuation (Figure 1). This can be triggered in metazoans by either a classic cleavage and polyadenylation factor PCF11 at PASs in introns (34,35), or by the Integrator complex in the proximity of promoters (36–41). Premature termination occurs also upon U1 depletion (42), U2-dependent splicing inhibition (43), heat shock (44) and CKD12 loss (45,46), possibly indirectly. Additionally, early termination occurs also on non-coding transcripts triggered by the Restrictor complex (47–49). The conceptual framework for the recent advances regarding metazoan transcription attenuation was laid down by fundamental yeast work on the Nrd1-Nab3-Sen1 (NNS) system. NNS controls the non-coding transcriptome (50) but also binds nascent RNA at protein-coding genes (51–53) and attenuates their transcription (51,53–59). Engaging in transcription and then attenuating/terminating this process prematurely appears energetically wasteful. However, the evolutionary conservation of this process in prokaryotes, yeast and metazoans, along with specific gene examples, highlights important regulatory roles for premature transcription termination (20,24,60,61). Recent structural studies shed light on the mechanisms of promoter-proximal termination of Pol II transcription by the human Integrator complex, which involves both steric changes and exonuclease activity, consistent with the unified termination model (62–64).

### The CTD code

The CTD of Pol II plays a regulatory role throughout all stages of transcription. This tail-like, flexible domain is composed of tandem repeats of a heptapeptide Y_1_S_2_P_3_T_4_S_5_P_6_S_7_ (Tyr1-Ser2-Pro3-Thr4-Ser5-Pro6-Ser7). The CTD undergoes many types of post-translational modifications, including phosphorylation, methylation, glycosylation, ubiquitylation and isomerization (65–67). The primary CTD modification is the phosphorylation of serine, threonine and tyrosine residues (68,69). Distinct patterns of CTD phosphorylation correlate with different stages of transcription, which forms the basis of a molecular language called the ‘CTD code’ (1). The most studied CTD marks are S5ph, which peaks during initiation, and S2ph marking Pol II productive elongation (70) (Figure 1). Y1, T4 and S7 phosphorylation marks are less studied and increase the diversity of the CTD code. Functionally, specific phosphorylation states of the CTD allow the binding of unique proteins. For example, S5 phosphorylation helps to recruit the capping enzymes and facilitates capping (71,72) and S7 phosphorylation is needed for the recruitment of the Integrator complex to small nuclear RNA (snRNA) genes (73,74). Y1 prevents binding of yeast termination factors and stimulates binding of elongation factor Spt6 *in vitro* (75), while S2 phosphorylation can recruit several 3′-end processing factors (76). Thus, CTD provides a binding platform for regulatory factors and processing machinery along the transcription pathway, modulated via the specific phosphorylation sites.

The effect of the CTD code varies among organisms and not all residues of the heptad contribute equally to gene expression and viability. For example, S2, T4, S7 and Y1 hydroxyl groups are dispensable for vegetative growth in fission yeast (72) but in metazoans S2, T4 and S7 are important for cell viability (74,77,78).

The number of heptad repeats present in a species, and the prevalence of non-consensus repeats (with altered amino acid sequence) increases higher up the evolutionary ladder. In *Saccharomyces cerevisiae* Pol II CTD contains 26 repeats, while in humans, it has 52. In yeast, the majority of repeats are consensus, while in mammals 31 of 52 repeats are non-consensus with most of them located in the distal part of the CTD and differing at the seventh position of the heptad (11). The CTD in *Drosophila melanogaster* is exceptionally variable, with only 3 of 45 repeats matching the consensus (10). There appears to be a correlation of increased complexity of the CTD code with increasing complexity of the organism. This might allow coordination of the expanding panel of CTD interacting partners that participate in the multiple gene expression pathways of higher eukaryotes (10,11). Interestingly, evolutionary analysis showed that the presence of a CTD in a species coincides with the presence of conserved histone H3 and H4 N-terminal tail sequence, implying that the so-called histone code and CTD code co-evolved allowing a functional cross-talk between them (11).

Intriguingly, Pol II CTD appears dispensable for transcription initiation and elongation, yet is required for transcription termination. It had been known for ∼35 years that the CTD is not necessary for initiation and elongation *in vitro* (79–81) but this was only recently confirmed in living human cells. In a study performed in the Roeder lab, an auxin-degron system for RBP1 subunit of Pol II was established. However, the auxin-induced degradation of RBP1 was incomplete, generating a CTD-less Pol II complex. This complex was elongation-competent and able to be released from paused state, but termination-incapable (82,83). In parallel, the Eick lab came to the same conclusions using a different experimental approach, developed originally in David Bentley’s lab (84). Endogenous Pol II was blocked with α-amanitin and simultaneously α-amanitin resistant RPB1 rescue vectors were expressed under the control of the Tet-off system (85). A control recombinant wild-type (WT) vector contained the human WT CTD sequence and rescued cell viability upon α-amanitin treatment. The tested human CTD-depleted Pol II (CTD-Δ5) contained only five repeats (1–3 and 51–52), essential for Pol II stability. Additionally, a previously studied CTD mutant (YFFF) was used, in which three-fourth of the Y1 residues were mutated (86). Neither CTD-Δ5 nor YFFF mutants could rescue cell viability, with the former showing a more severe phenotype. Surprisingly, both mutants were able to initiate and elongate transcription, yet showed a pervasive transcription termination failure. Previously, CTD-Δ5 experiments performed on a smaller scale in the same lab detected only defective initiation (87,88). Interestingly, both mutants were characterized by a loss of Pol II interactions with the Mediator and Integrator complexes. The authors proposed a model in which CTD-depleted Pol II has a reduced rate of DNA entry, but once Pol II engages in transcription, the process becomes extremely pervasive, leading to a global loss of termination (85).

In summary, Pol II CTD evolved at the same time as conserved histone N-terminal tails, and both of them undergo extensive post-translational modifications that form a molecular ‘code’, which is ‘read’ by different binding proteins. Pol II CTD is essential for eukaryotic life, and is in particular necessary for the last part of the transcription cycle, that is transcription termination.

### A mark for transcription termination: phosphorylation of Pol II CTD threonine 4

Despite a substantial progress in the transcription field, termination still remains insufficiently studied. One of the reasons underlying this, especially in the genome-wide context, is the lack of appropriate tools. A breakthrough in transcription studies was achieved with the advent of nascent transcriptomics methods, such as GRO-seq, PRO-seq, chromatin-bound RNA-seq, mNET-seq, TT-seq and POINT-seq (43,89–92). Currently used techniques can be divided into two types: single-nucleotide-resolution methods measuring exact positions of Pol II, which are particularly sensitive to Pol II pausing (e.g. PRO-seq and mNET-seq); and methods measuring longer fragments of nascent RNA, which are less sensitive to Pol II pausing (e.g. TT-seq and POINT-seq).

**Figure 2.**
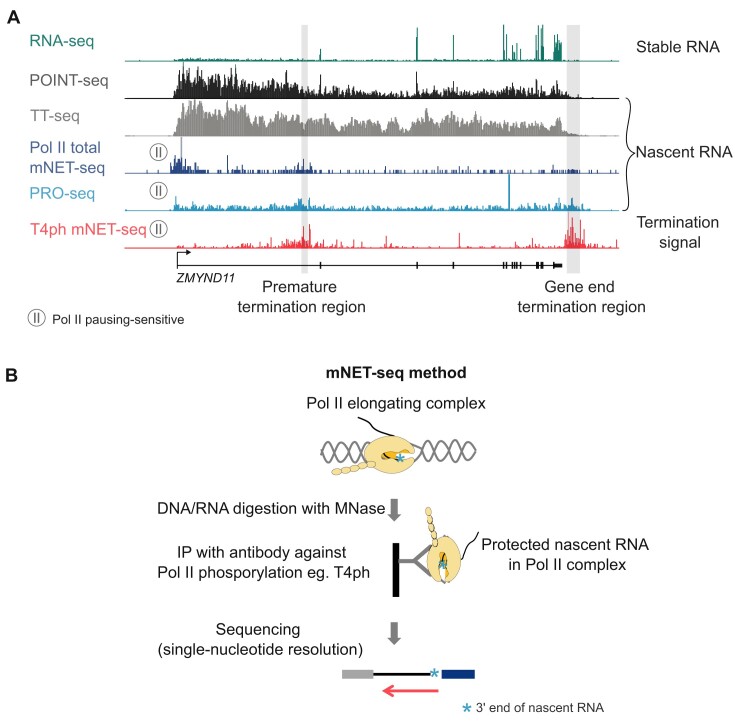
Comparison of various nascent transcriptomics techniques. (**A**) *ZMYND11* genomic profile with tracks generated using different transcriptomic techniques. Besides RNA-seq (top panel) all other techniques measure nascent RNA. For simplicity only + strand signal is shown. (**B**) General outline of the mNET-seq method.

However, for all of the discussed methods it remains hard to determine the genomic windows in which Pol II termination occurs based on signal of total nascent RNA synthesis – it requires finding an optimal threshold between the gradually decreasing signal at the end of genes, and the intergenic signal of basal pervasive non-coding transcription of the genome (Figure 2A, black, gray and blue signal). In the case of premature termination within gene body, this becomes even more challenging. In contrast, quantifying the appearance of a termination mark is more robust. Here, we argue that Pol II phosphorylated on threonine 4 provides such a useful mark for Pol II entering the terminal pausing mode. This is especially clear when assayed with the sensitive, strand-specific and single-nucleotide-resolution mNET-seq method [Figure 2A (red signal) B].

In the current literature, at least 12 different abbreviations exist for CTD threonine 4 phosphorylation (Table 1), making the mark poorly searchable in databases. Although the form ‘Thr4P’ has been the most widely used (in ∼20% of papers), this review adopts the form ‘T4ph’, which is consistent with the Brno nomenclature for histone modifications that has been uniformly adopted by the chromatin field since 2005 (93). We believe that such consistency in nomenclature could be beneficial, especially since many transcription studies involving Pol II CTD also reference and even involve histone modifications.

**Table 1.** Existing abbreviations of Pol II CTD threonine 4 phosphorylation found in literature

Abbreviation	No of papers using abbreviation	References	Animals	Yeast
Thr4P	12	(13,86,94–103)	Yes	Yes
Thr4-P	10	(11,65,77,104–110)	Yes	Yes
pThr4	5	(111–115)	Yes	Yes
P-Thr4	4	(78,116–118)	Yes	Yes
T4P	4	(119–122)	Yes	No
T4ph	2	(24,123)	Yes	No
Thr4p	2	(32,124)	Yes	Yes
Thr4-PO2	2	(125,126)	No	Yes
T4-P	1	(66)	Yes	Yes
Thr(P)-4	1	(127)	No	Yes
Phospho-Thr4	1	(128)	Yes	Yes
T_4_-P	1	(129)	Yes	Yes

This review focuses on Pol II CTD threonine 4 phosphorylation (Pol II CTD-T4ph) in metazoans, particularly highlighting the existing literature and re-analyzing genomic datasets generated up to date that demonstrate it is a unique mark of transcription termination genome-wide.

## Materials and methods

### Datasets used in this study

**Table:** 

Data type	Publication	GEO accession
RNA-seq	(130)	GSM7179805
mNET-seq (T4ph)	(34)	GSE123105
mNET-seq (total Pol II, S5ph, S2ph, Y1ph, S7ph and T4ph)	(121)	GSE81662
mNET-seq (T4ph)	(120)	GSM2976656
POINT-seq	(43)	GSM4826609
TT-seq	(49)	GSM7119959
PRO-seq	(131)	GSM2692352
ChIP-seq	(77)	GSM920945
ChIP-seq	(100)	GSM2686260

### Reference genomes and annotation

Hg38/GRCh38 was used as the reference genome. GENCODE release 40 was used for annotation (basic annotation was chosen). For the analysis, we selected 10 387 protein-coding genes, that did not overlap with another annotated gene on the same strand and had a 3′-end isolated by at least 6 kb from the downstream annotated gene on the same strand.

For *D. melanogaster*, dm3 was used as a reference genome. Annotation from UCSC accessed through AnnotationHub package (Morgan, M. and Shepherd, L. (2024) AnnotationHub: Client to access AnnotationHub resources) was used. We extracted 15 682 transcripts that were next used to plot a metagene representation of chromatin immunoprecipitation followed by sequencing (ChIP-seq).

### Definition of termination windows

To define termination windows genome-wide, we used five biological replicates of T4ph mNET-seq from two different publications: three biological replicates from (34) and two biological replicates from (118). For each study, we created an average T4ph mNET-seq file using wiggletools mean. Then, single-nucleotide resolution files were manipulated in R: reads were resized to 150 bp and then bigwig files were converted to bedGraphs. To find the enriched regions, macs2 bdgbroadcall function (128) was called on bedGraph files, using default parameters. We selected only the enriched regions that at least partially overlap in two average files and then merged the overlapping ranges [see GenomicRanges reduce function (129)]. We ended up with 13 488 termination windows, then we divided them into three groups based on their localization:

1. Gene-end termination windows: overlapping predefined + 6-kb region after annotated PAS of preselected protein-coding genes, *n* = 3566.
2. Premature termination windows: falling fully into gene bodies of preselected protein-coding genes, *n* = 1376.
3. Other termination windows: excluded from two categories, either intergenic or normal/premature for genes that were excluded from the analysis, *n* = 8546.

### Metagene profiles

Metaplots and heatmaps were plotted using deeptools (130). For all mNET-seq metagenes/heatmaps and other nascent RNA datasets, we used only files coming from the forward strand; consequently, we used reference genomic regions/termination windows also from the forward strand. *N* genes = 5247, *n* gene-end terminators = 1837 and *n* premature terminators = 715.

To scale datasets to 1, unscaled files were first used to compute matrices by computeMatrix scale-regions function by deeptools, then plotted using plotProfile function with –outFileNameData specifying output .tab file with numeric data underlying the plot. Using this .tab file, we extracted maximum value for each bigwig file visible on a plot. Then, we used this maximum value to calculate scaling factor = 1/max. Having the scaling factors, we multiplied scores of each bigwig file in R. Finally, the profiles/heatmaps were plotted for the second time using scaled bigwigs as an input.

### Other analyses

All datasets built on other versions of reference genomes, were lifted to hg38 using CrossMap tool (131).

To calculate PAS/TSS ratio, 200-bp windows either ending at annotated PAS, or starting at annotated TSS, were defined for all preselected genes (*n* = 10 387). Then, sum of the signal for each Pol II CTD modification (80) was calculated in these windows. Then, log2(PAS/TSS) was plotted.

To plot average T4ph mNET-seq signal based on genes activity, average total Pol II and T4ph mNET-seq signal (118) was calculated in the gene body + 5 Kb of selected genes. Based on average total Pol II mNET-seq signal, genes were divided into five equal groups (*n* = 2077) and labeled based on their activity. Then, average T4ph mNET-seq signal was plotted in these five groups.

## POL II CTD-T4ph in yeast

A recent excellent review from the Ansari lab thoroughly covers T4ph functions in yeast (132). Here, we merely summarize findings from yeast regarding the process of transcription termination.

### T4ph metabolism, functions and levels in yeast

In a systematic study, Nemec *et al.* showed that in *S. cerevisiae*, 11 kinases phosphorylate T4 *in vitro* at least four-fold above background. However, inhibiting only four of these enzymes resulted in diminished levels of T4ph *in vivo* (109). T4 kinases play important roles in nutrient or phosphate sensing as well as regulation of response to environmental stress (133–136). Importantly from the perspective of this review, the most active T4 kinase, Hrr25, was shown to be significantly enriched at the 3′-ends of small nucleolar RNAs (snoRNAs), and to a lesser extent across the length of protein-coding genes. Selective inhibition of Hrr25 resulted in termination defects at snoRNA transcripts and elongation defects at protein-coding genes (109).

From the physiological point of view, the presence of threonine 4 in CTD repeats is not crucial for viability of yeast – in *Schizosaccharomyces pombe*, only S5 is essential (72). In *S. cerevisiae*, T4 substitution with alanine or valine is viable even when coupled with a S7A mutation (137) in rich media but becomes relevant in low-phosphate or galactose-containing medium. In particular, T4 stimulates eviction of histone variant Htz1 from gene promoters, therefore inducing genes involved in phosphate and galactose metabolism (114). Mirroring these results, RNA-seq analysis of poly(A)+ RNA from T4A mutants showed diminished levels of genes encoding proteins related to phosphate uptake in *S. pombe* (138). Thus, CTD-T4ph is dispensable for yeast viability but is involved in phosphate metabolism.

In 2016, two mass spectrometry studies focused on quantifying CTD phosphorylated heptads *in vivo* in *S. cerevisiae* came out side-by-side from the laboratories of Dirk Eick and Stephen Buratowski. Both groups were in agreement that heptads are mainly mono-phosphorylated and the highest levels of phosphorylation in yeast occur on S5, followed by S2. However, Schüller *et al.* found that T4ph abundance is around 20% (125), while Suh *et al.* detected almost two orders of magnitude lower levels of T4ph compared with S5ph (105). Recent work from the Buratowski lab utilized an improved experimental system, and confirmed that in *S. cerevisiae* most CTD phosphorylation occurs on S5 and S2. However, T4ph was undetectable (139). These findings highlight the need of further work to clarify the phosphorylation levels of specific CTD residues in various species.

### Threonine 4 phosphorylation localization and T4 requirement for transcription termination in yeast

In *S. cerevisiae*, T4ph is present within the gene body of protein-coding genes (75) as well as past the PAS (101). In contrast, for non-coding RNA most of the signal is enriched at the 3′-end. T4-dependent non-coding snoRNAs show significant read-through in T4A mutants in *S. cerevisiae* (140). However, only selected groups of snoRNA are affected by the T4A mutation, namely snoRNA bearing high-T4ph and low-S2ph levels at their 3′-end in WT cells. On the contrary, snoRNAs that undergo normal termination in T4A mutants exhibit S2ph enrichment instead of T4ph at the 3′-end. Therefore T4ph has not only a different role between RNA types, but even within a given class of RNA (140). This adds to the elusiveness of this mark in yeast. In fission yeast *S. pombe*, the T4A mutant also affects termination of non-coding RNA (123). Dis2 dephosphorylates T4ph on the CTD of Rpb1 facilitating transition of Pol II from elongation to termination. A depletion of Dis2 leads to delayed Pol II dissociation and a global enrichment of T4ph at the 3′-ends of genes (141). Generally, T4ph enrichment has been observed at termination sites on most protein coding genes in both yeast species (102,141).

### Termination factors binding to phosphorylated threonine 4 in yeast

Consistent with other CTD marks, T4ph can mechanistically function as a binding platform for proteins interacting with Pol II. In *S. cerevisiae*, affinity enrichment followed by mass spectrometry revealed that CTD containing T4ph interacts with Rtt103 (102,105), and this interaction is sensitive to the T4A mutation (140). Rtt103 is a termination factor which functions together with the exonuclease complex Rat1/Rai1 on polyadenylated transcripts (26,142). Rtt103 can also directly bind to S2ph, yet it is only recruited downstream of the PAS. This specificity is controlled in part by Y1ph, presumably through blocking Rtt103 binding upstream of the PAS (102). Native sequential immunoprecipitation (IP) strategy facilitated the purification of Pol II complexes enriched for different phosphorylated CTD residues. As expected, the S2ph antisera were able to compete with Rtt103 for CTD binding, and the S5ph antisera were not effective in competition. Consistent with the mass spectrometry data, T4ph antisera were also able to compete with Rtt103 for CTD binding, indicating both residues likely regulate the interaction between Rtt103 and the Pol II CTD (102). NET-seq and ChIP-seq experiments demonstrated that Rtt103p is co-localized with the T4ph mark downstream of the PAS. Deletion of the Rtt103p protein, as well as expression of RPB1 T4V CTD mutant, causes similar RNA Pol II pausing defect downstream of the PAS (113). Additionally, Vasilieva lab has demonstrated in *S. pombe* that the CTD-interacting domain (CID) of transcription termination factor Pcf11 binds to S2ph and T4ph with similar affinity (141). The CIDs of other termination factors tested by the authors (*S. pombe* Seb1, *S. cerevisiae* Nrd1 and Rtt103) also interacted with both S2ph- and T4ph-modified CTD peptides, although in this case the affinity to T4ph was significantly lower.

The conclusion from the above studies is that threonine 4 and its phosphorylation are not essential for viability in yeast. Even so, for this eukaryotic taxon, threonine 4 phosphorylation is associated with transcription termination and promotes correct localization of the process. Furthermore, several yeast termination factors are able to bind Pol II CTD-T4ph.

## POL II CTD-T4ph in metazoans

### CTD threonine 4 is required for viability of metazoan cells

The first study on the importance of T4 phosphorylation in animals was conducted by Hsin *et al.* in the Manley lab on the chicken DT40 cell line with a tetracycline-repressible complementary DNA of the human Rpb1. This system allowed for genetic complementation experiments using either WT CTD repeats or CTD repeats with T4 mutated to V (T4V). T4V cells lost viability after 24 h, but the total transcription level and synthesis of polyadenylated mRNAs were not significantly affected, decreasing only by 20%. The abundance of various Pol II transcripts in T4V cells remained close to WT, except for non-polyadenylated replication-dependent histone mRNA which were reduced to 15%. However, nuclear run-on analysis revealed that transcription of histone genes was not reduced in T4V cells. It was concluded that T4V mutation led to defective U7-dependent 3′-end processing and promoted utilization of cryptic downstream PASs (78).

Expression of Pol II with its CTD mutated on threonine 4 also affects the viability of human cells (as reported by Hintermair *et al.*). A drastic decrease in HEK293 cell viability and proliferation rate was observed at 4 days after cell transfection with amanitin-resistant mutant CTD Rpb1 followed by depletion of endogenous Rpb1. The growth of S2A and S5A mutants was the most impaired, T4A substitution had shown similar results, while T4S, as well as S7A substitutions, had a milder effect on cell viability. Similar results were also demonstrated for the human B-cell line Raji (77).

### The level of threonine 4 phosphorylation in human cells

To date, only one mass spectrometry study measured the levels of phosphorylated amino acids on human CTD – the work of Schüller *et al.*, mentioned earlier in the corresponding yeast paragraph. The authors found that in the Raji cell line phosphorylated S2 and S5 comprised 38% and 40% of the total phospho-counts in mono-phosphorylated repeats, respectively. Heptad repeats with phosphorylated T4 had relative abundance of 15%, while the frequency of repeats with phosphorylated Y1 and S7 did not exceed 5%. When analyzing double-phosphorylated heptad repeats, all possible double-phospho combinations were detected within single heptads. However, the total counts of double-phosphorylated repeats were 30 times lower compared with mono-phosphorylated repeats. It suggests that the concurrent presence of two phosphosites within a single repeat is a rare event in human cells *in vivo*. Phosphorylated T4 preferentially co-occurred with S2 and S5 phosphorylations (125).

### CTD T4 kinases in animals

Hsin *et al.* proposed CDK9 as a kinase responsible for T4 phosphorylation in chicken DT40 cells. They demonstrated that this phosphorylation was sensitive to the CDK9/P-TEFb inhibitors DRB and flavopiridol, and that the level of histone mRNA was reduced in cells treated with these inhibitors to a level comparable to T4V cells (78).

An important new tool was the generation of a specific monoclonal antibody for CTD-T4ph in the Eick lab (77). This enabled the confirmation of the existence of T4ph *in vivo* in human cells. Notably, this modification occurs specifically on hyper-phosphorylated Pol II, designated as Pol II0. A panel of 81 recombinant S/T kinases was screened for specificity, initially on a CTD peptide, and subsequently on purified full-length Pol II. Two positive kinases identified in this screen were Plk3 and Plk1. Plk kinases are usually strictly associated with specific phases of mitosis; however, Plk3 might be present in cells throughout the cell cycle, therefore could potentially phosphorylate CTD-T4 outside of mitosis (77). Importantly, in the same study it was found that S2ph is required for T4 phosphorylation. This suggests an alternative explanation for the CDK9/P-TEFb inhibitors effect as suggested by Hsin *et al.* (78): CDK9 might not be a direct kinase for T4, but rather be indirectly required for T4ph *in vivo*.

In a follow-up study, Hintermair *et al.* examined the interaction of Pol II with Plk1 in mitotic cells by co-IP experiments. Plk1-specific antibodies co-precipitated a small fraction of the mitosis-specific, even more highly hyper-phosphorylated Pol II00 form of nocodazole-treated cells and *vice versa*, the T4ph-specific antibody precipitated Plk1. However, *in vitro* phosphorylation of CTD T4 residues by Plk1 and Plk3 did not shift the Pol II to the II00 form. The authors hypothesized that additional, yet unidentified modifications contribute to the transition of T4 phosphorylated Pol II0 to the II00 form *in vivo* (104). Considering that 11 kinases can phosphorylate T4 in yeast (as mentioned above), further T4 kinases might be identified in animals in the future.

### The use of antibodies and T4ph detection

Eick lab generated two monoclonal antibodies against T4ph, 6D7 and 1G7, and the clone they considered superior, 6D7, is now commercially available from Active Motif (RRID: AB_2750848). To our knowledge, most publications on T4ph to date, except for the two mentioned below, have used the 6D7 clone. It is also most characterized one, with well demonstrated specificity to T4ph (77,143). However, the first paper introducing this tool, Hintermair *et al.* 2012, demonstrated using *in vitro* generated doubly phosphorylated peptides that neighboring phosphorylation on S2 or S5 could interfere with antibody recognition. This lead to the concern that T4ph antibodies might detect a decrease in S2ph and/or S5ph rather than an increase of T4ph at some genomic locations. Importantly, subsequent mass spectrometry by Schüller *et al.* showed that such occlusion is very unlikely to occur *in vivo*, since single heptad repeats (both human and yeast) typically carry only one phospho-mark *in vivo* (125). In particular, according to mass spec measurements, T4 phosphorylation is 20 times more abundant as single modification compared with doubly phosphorylated peptide. 6D7 shows an expected immunofluorescence pattern – following global Pol II as well as S2ph distribution in interphase cells (77). Surprisingly, subsequent immunofluorescence analysis by the same group found that, on mitotic chromosomes, 6D7 and other anti-T4ph clones exhibited a different pattern compared with other CTD phosphorylations, localizing to centrosomes (104). Because this pattern was exclusive to mitosis yet consistent across various cell lines and antibodies, the authors interpreted it as true mitotic T4ph localization. However, it cannot be excluded that 6D7 cross-reacts with a mitosis-specific phosphorylation of a centrosomal protein.

In our experience, the primary limitation of 6D7 antibody is its poor performance and non-linearity in western blot assays; therefore, we strongly advice against using it in this application. If used despite this recommendation, it should be limited to qualitative, and not quantitative, assessments. Instead, Manley, Proudfoot and Calvo labs have successfully used rabbit polyclonal antibody NBP1-49546 for western blot in published work in yeast, fly and human (34,78,115,144).

Additionally, two published studies used a rabbit monoclonal antibody #26319 from Cell Signaling Technology (100,116). This antibody, while less characterized, appears to exhibit very similar behavior to clone 6D7. It is specific for T4ph in dot blot assays, with artificially co-phosphorylated S2 and S5 in the same heptad partially blocking recognition. Additionally, it shows a distinct enrichment at metazoan gene ends, as demonstrated by ChIP and CUT&RUN, mirroring enrichments found by 6D7.

A recently published study implies that T4ph levels at the TSS are hugely underestimated due to S5ph occlusion (143). We do not find the data convincing as (i) the non-linear 6D7 antibody was used by the authors for western blot quantifications; (ii) Ssu72 phosphatase specificity was not tested in a cell lysate and the distribution of other phospho-marks upon Ssu72 treatment has not been checked; (iii) a cytoplasmic protein was used as loading control and western blot normalization factor in nuclear extracts; and (iv) 20-fold more single-phosphorylated rather than double-phosphorylated T4ph peptides were found in mass spectrometry (125). In light of the above issues, an antibody should be generated to verify the existence and function of a putative T4phS5ph double-phosphorylated Pol II bound to the TSS.

It is evident that the quality of the antibodies used is crucial for the robustness of the resulting research, particularly given the experience of the chromatin community in this regard. Therefore, it is highly advisable to characterize the currently used antibodies in greater detail and to develop additional tools for studying T4ph. For example, new nanobodies or polyclonal antibodies might be able to recognize T4ph in the context of both single and doubly phosphorylated heptads. Alternatively, antibodies could be raised specifically to recognize the doubly phosphorylated heptads. Further on, antibody-independent method development, such as mass spectrometry-based, is also greatly needed to study T4ph with more robustness.

## POL II CTD-T4ph as a marker of transcription termination of protein-coding genes in metazoans

### Pol II CTD-T4ph is enriched at 3′-ends of protein-coding genes in metazoans

**Figure 3.**
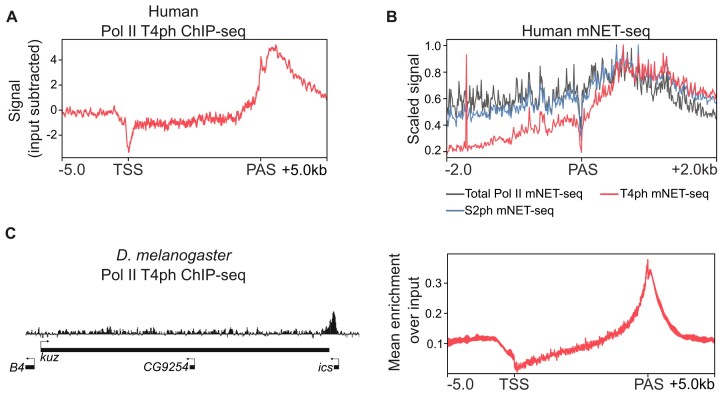
Pol II CTD-T4ph modification measured by ChIP-seq in human (77) and *D. melanogaster* (100) cells and its position in comparison with S2ph measured by mNET-seq (118). (**A**) Metagene profile for Pol II CTD-T4ph ChIP-seq (77) in human (*n* = 10 387). (**B**) Metaplot of total, S2ph and T4ph mNET-seq signal (118) ± 2 kb from PAS (*n* = 5247). (**C**) Snapshot of a genomic locus demonstrating gene-end enrichment of T4ph in ML-DmBG3-c2 (BG3) *D. melanogaster* cells detected by ChIP-seq (100) (left panel) and metagene profile (right panel) for Pol II CTD-T4ph ChIP-seq (*n* = 15 682).

A common method to study the connection between CTD modification and transcription is ChIP-seq. In the landmark study mentioned above, Hintermair *et al.* performed ChIP-seq experiments on the human B-cell line Raji to identify the steps of the transcription cycle involving T4 phosphorylation (77). Signals for T4ph were low but detectable at TSS for a few genes, slightly rose in the body of genes, but strongly increased at the 3′-end of genes, reaching a maximum between 500 and 2000 nucleotides downstream of the PAS. Here, we re-analyzed the datasets provided with this paper to confirm the global association of T4ph downstream of the PAS in protein-coding genes (Figure 3A).

Inspection of individual genes by the authors (*HSPD1, PTMA* and *NCL*) revealed significant differences in the local occurrence of the S2ph and T4ph at the 3′-end of genes. While S2ph signals increased concomitantly with Pol II levels at gene 3′-ends, the increase of T4ph levels was delayed to several hundred nucleotides later. This suggests that phosphorylation of CTD S2 precedes the phosphorylation of T4 at the 3′-end of genes in human cells (77). To corroborate this observation using a higher resolution technique, we re-analyzed mNET-seq data for S2ph and T4ph conducted on human HeLa cells. Notably, this single-nucleotide-resolution methodology confirms the delayed phosphorylation of T4 compared with S2, which was not previously shown using this technique (Figure 3B).

In a study involving *D. melanogaster* ML-DmB3-c2 (BG3) cells, ChIP-seq patterns were explored after IP with antibodies against different phosphorylated CTD residues. The study investigated the role of cohesion-mediated recruitment of Polycomb repressive complex 1 in the transcriptional control of active genes (100). Here, both in metagene analysis and for the specific gene *headcase*, ChIP-seq patterns for CTD phosphorylation were as follows: S5ph signals peaked downstream the TSS and then gradually decreased in the gene body; S7ph mark increased downstream the TSS, had a low level across the gene body and then raised again past the gene end; S2ph levels were high across the whole gene consistent with a role in transcription elongation; and T4ph mark was low over the gene body but peaked just after the PAS (100). Our re-analysis of the published datasets from Pherson *et al.* confirms this enrichment of T4ph at gene ends (Figure 3C).

Therefore, data from human and *D. melanogaster* cells consistently show T4ph enrichment downstream of PAS, further supporting a link between this mark and transcription termination in metazoans.

### Pol II CTD-T4ph measured by mNET-seq allows for a better characterization of termination regions

While ChIP-seq clearly shows T4ph enrichment at gene ends, it does not distinguish which strand is transcribed by Pol II in a given locus, and is limited by an average resolution of 200–500 nt. In contrast, data obtained using mNET-seq technology have three major advantages: (i) strand specificity, (ii) single-nucleotide resolution and (iii) lower background (91), therefore we chose to focus on this method.

**Figure 4.**
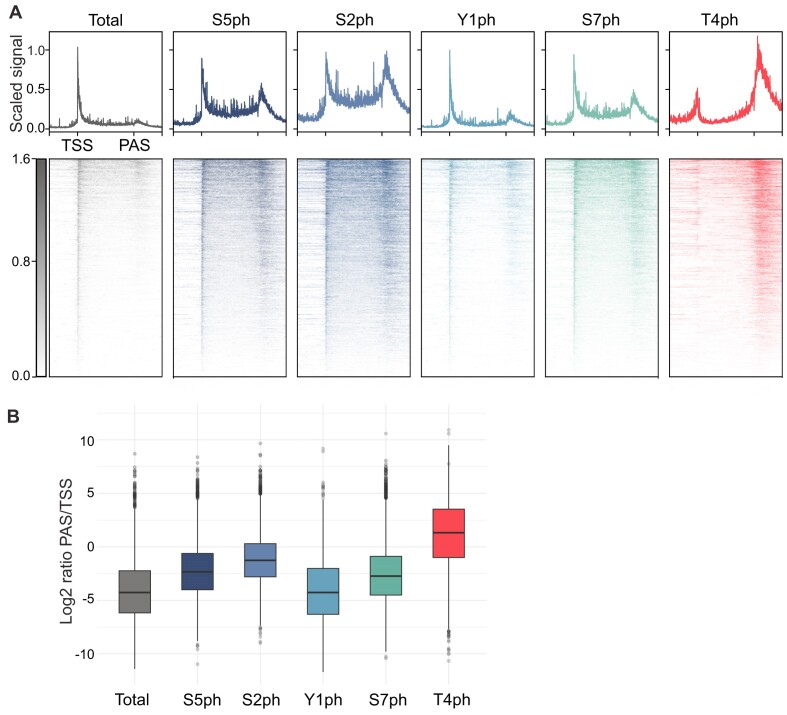
Pol II CTD phosphorylation mNET-seq patterns across active human protein-coding genes. Data from (118). (**A**) Metagenes and heatmaps representing the profiles of mNET-seq using antibodies against total Pol II (gray) and phosphorylated forms: S5ph, S2ph, Y1ph, S7ph (shades of blue and green) and T4ph (red), *n* = 5247. Regions upstream of TSS/downstream of PAS are 5-kb long. (**B**) Box plot ratio of mNET-seq signal at PAS/TSS for each modification (*n* = 10 387).

Genomic profiles of Pol II phosphorylated at different sites, as measured by mNET-seq in human HeLa cells by Schlackow *et al.* (118) are shown in Figure 4A. By both metagene and heatmap analysis, we recapitulated the finding that T4ph is enriched mostly after PASs. The peak localizes around 1–2 kb after annotated PAS. Our further analysis additionally reveals that T4ph is the only phospho-mark that is enriched in PAS regions compared with TSS regions (Figure 4B). This is complementary to and consistent with the observation made by Schlackow *et al.*, in their Supplementary Figure S1 showing that T4ph is characterized by the highest termination index among all analyzed CTD phosphorylations in human cells.

**Figure 5.**
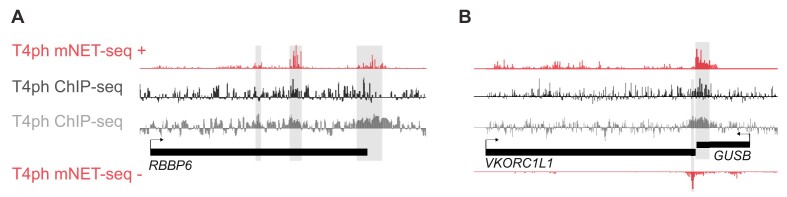
Comparison between T4ph ChIP-seq conducted on Raji and HEK293 cells [dark gray (77) and light gray (143), respectively] and mNET-seq done on HeLa cells [average signal from three biological replicates (34)]. Shaded areas correspond to termination windows defined by our method. (**A**) Snapshot of *RBBP6* gene that undergoes premature transcription termination. There is only transcription on the + strand in this region. (**B**) Snapshot of two close by convergent genes (*VKORC1L1* and *GUSB*). Please note mNET-seq can differentiate whether the T4ph signal is coming from the gene encoded on + or – strand, which is not possible with strand-agnostic ChIP-seq.

mNET-seq is the most sensitive and high-resolution method for detecting T4ph-modified Pol II, which becomes especially apparent when analyzing termination regions of individual genes (Figure 5). The strand specificity of mNET-seq is particularly advantageous when examining the example on Figure 5B, where distinct termination windows from two genes on opposite strands merge into a single ChIP-seq peak. Importantly, the results obtained by mNET-seq are consistent with those assessed by ChIP and ChIP-seq performed in human cells (32,77,116) (Figure 3A, and data in original papers). Moreover, the same pattern is evolutionarily conserved, with T4ph localizing to the ends of *D. melanogaster* genes as measured by ChIP-seq (100) (Figure 3C).

To conclude, T4ph enrichment at protein-coding gene ends has been observed with the use of different methods (both native and cross-link dependent), in multiple animal cell systems, and with the use of different antibodies.

**Figure 6.**
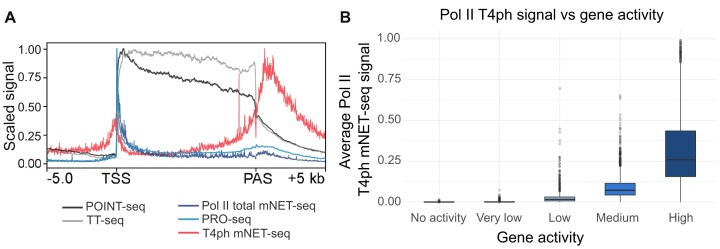
T4ph mark is associated with ends of active protein-coding genes. (**A**) Metagene profile of POINT-seq, TT-seq, PRO-seq, Pol II total mNET-seq and T4ph mNET-seq (*n* = 5 247). (**B**) Average T4ph mNET-seq signal in the gene body + 5 Kb of protein coding genes. Genes were divided on five equal groups (for one group *n* = 2 077) based on average total Pol II (modification-independent) mNET-seq signal [data from (118)].

The question arises whether this association is global or gene-specific, and whether it occurs on active genes undergoing transcription elongation. The analysis shown on the heatmap in Figure 4A following (118) and metaplot with nascent RNA signal (Figure 6A) strongly suggests that T4ph is globally associated with termination regions downstream of the PAS. To reliably show termination sites, a marker should be not only visible at the ends of the genes but also associated only with active transcriptional units. To exclude the possibility that T4ph mark is associated with inactive genes, we divided genes into five equal groups based on the average signal of total mNET-seq in their gene bodies (Figure 6B). We observed that genes with the highest activity show the highest T4ph signal. Genes that are not active are not associated with this Pol II modification. This confirms that T4ph is associated with actively transcribed protein-coding genes, and increases with gene activity. While aligning with expectations, this was not previously demonstrated using any genomic methods.

Other evidence that T4ph follows gene activity in human cells comes from earlier immunofluorescence experiments (77). There, Supplementary Figure S2 demonstrates that T4ph staining, similarly to S2ph, followed general Pol II staining, while S5ph and S7ph staining showed patterns indicative of more focused, preferential phosphorylation.

In summary, T4ph appears to associate indiscriminately with active genes, with no apparent subset of specific target genes, and localizes predominantly at the end of genes.

### Unusual locations: Pol II CTD-T4ph at TSSs and gene body premature termination sites

Pol II CTD-T4ph is highly enriched in regions downstream of the PAS, noticeable both on averaged genomic signal (Figures 3A and 6A), and also clearly visible on individual genes, as seen on the example of *ZMYND11* (Figure 2A). However, when aggregated on a metagene or heatmap (Figures 4A and 6A), a lower level of enrichment can be also observed at TSSs. This TSS enrichment is typically not convincing on individual genes (Figures 2A and 5). TSS-proximal threonine 4 phosphorylation signal is likely connected to TSS-linked premature termination. High levels of Pol II turnover close to TSS in metazoans have been demonstrated in landmark papers using single molecule footprinting in fly (145) and computational modeling of Pol II kinetics measured by FRAP in human cells (146). Interestingly, TSS-linked threonine 4 phosphorylation signal appears prior to productive elongation signal as visualized by TT-seq and POINT-seq (Figure 6A), which fits with the idea that it marks unproductive terminating Pol II.

Besides the TSS, protein-coding gene body might appear to be another unusual localization for a termination mark. However, existence of gene-body premature termination in metazoans is well supported. The strand-specificity and high resolution of T4ph mNET-seq signal enabled, for the first time, the direct detection of premature termination (Pol II terminal pausing) sites within the body of protein-coding genes in humans (34). Premature termination downstream of the TSS occurs mainly in introns, is typically linked with a PAS and is prominent and easily detectable with T4ph mNET-seq when looking at the single gene level (Figures 2A and 5A). In contrast, its variable localization across different genes makes it difficult to detect in metagene analyses.

### Termination windows, defined as Pol II CTD-T4ph-enriched regions measured by mNET-seq, are characterized by a decrease in nascent RNA signal and increased Pol II pausing

We sought to characterize termination windows downstream of PAS of protein-coding genes, as well as premature termination windows occurring within gene bodies. We performed new analyses using data from five biological repeats of T4ph mNET-seq from two different studies (34,118). The analysis algorithm is described in the methods section. In short, we extended the single-nucleotide mNET-seq position to 150 bp, and used MACS2 for ‘calling’ T4ph-enriched regions, which we named termination windows. To exclude artifacts due to quantifying signal from closely spaced tandem genes, we only analyzed active genes that have no close downstream neighbor on the same strand. We then classified termination windows as ‘premature’ if they overlapped with the gene body of protein-coding genes, and ‘gene end’ if they were localized between the PAS and 6 kb downstream (region where transcription terminates for most human genes). In this way we obtained 1376 ‘premature’ and 3566 ‘gene end’ termination windows for downstream analysis.

**Figure 7.**
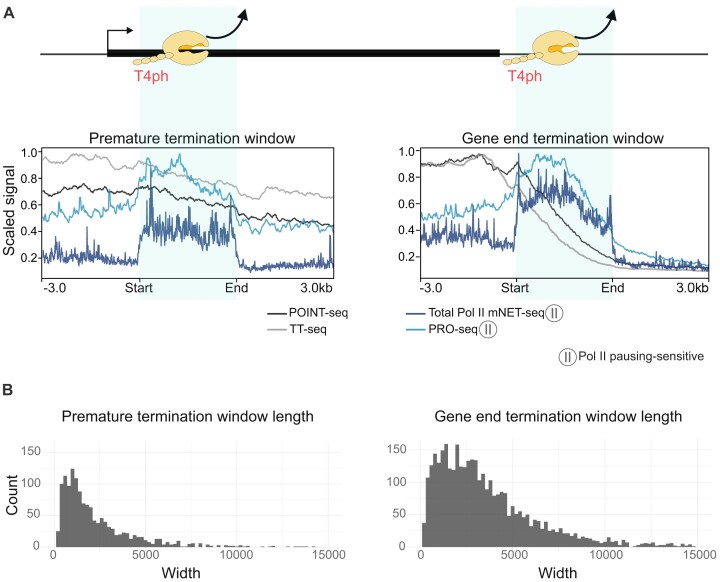
Termination windows characteristics (nascent RNA level, Pol II pausing and window length). (**A**) Nascent RNA signal and Pol II pausing in isolated premature (left panel; *n* = 715) and gene-end (right panel, *n* = 1837) termination windows. (**B**) Length of termination windows depending on their localization: premature (left panel) and gene end (right panel), *n* premature = 1376, *n* gene end = 3566.

We then plotted POINT-seq, TT-seq, PRO-seq and total Pol II mNET-seq signal over gene end and premature termination windows. PRO-seq and mNET-seq are nascent transcriptomic methods that measure Pol II positions with single-nucleotide resolution, and are particularly sensitive to RNA Pol II pausing. POINT-seq and TT-seq measure, respectively, entire or relatively long fragments of nascent RNA molecules – and are due to this less pausing sensitive. As expected, gene end termination windows correspond exactly to the genomic coordinates in which POINT-seq and TT-seq signals decrease strongly, showing a steep slope. This is in contrast to the flat profiles just upstream and downstream of the termination window (Figure 7A, right panel, black and gray lines) and indicates that cessation of nascent RNA production occurs in termination windows defined by the presence of T4ph. On the other hand, PRO-seq and total mNET-seq signals are enriched in the termination windows, compared with the regions upstream and downstream, indicating Pol II pausing in these regions (Figure 7A, right panel, light and dark blue lines, respectively). This finding is consistent with the necessity of Pol II pausing prior to its release from the DNA template. Importantly, both PRO-seq and total mNET-seq levels downstream of the termination window are markedly lower compared with the region upstream of the termination window. Therefore, Pol II molecules leave the DNA template within the termination window defined by T4ph enrichment.

In premature termination windows, POINT-seq and TT-seq profiles shift from nearly flat upstream to a noticeable slope at the termination window (Figure 7A, left panel, black and gray lines, respectively). This drop in transcript levels is less pronounced than in gene-end termination windows, which is consistent with only a fraction of transcripts terminating prematurely and others being synthetized until the regular gene end. PRO-seq and mNET-seq signal show a clear increase in premature termination windows (Figure 7A, left panel, light and dark blue lines, respectively), similar to gene-end terminators, again consistent with Pol II pausing requirement for transcription termination.

Interestingly, premature termination windows, with a median length of 1559 bp (Figure 7B, left panel), are generally almost two times shorter than gene end termination windows, which have a median length of 2900 bp (Figure 7B, right panel).

To summarize, all commonly used nascent transcriptomics techniques confirm that T4ph-enriched regions show the expected features of transcription termination windows, in which Pol II undergoes terminal pausing prior to leaving the DNA template. Our new approach allows now for the first time to determine genome-wide specific transcription termination windows localization in cells.

However, our current approach has two shortcomings that should be addressed in the future: (i) it does not take advantage of the single-nucleotide resolution of mNET-seq, and (ii) in some cases we observe a fragmentation of termination windows, which we think does not reflect the biological situation.

### Non-coding transcription termination of lncRNA is fundamentally different, and T4ph signal spreads across the whole transcription unit

In contrast with protein-coding genes, long non-coding RNA (lncRNA) generally do not show high T4ph 3′-end association, and instead, the T4ph profile is spread across the whole transcriptional unit. Proudfoot lab compared T4ph-mNET-seq profiles and their association with transcription termination and 3′-end processing on protein-coding and lncRNA genes in human cells (118). Protein-coding metagene analysis, as well as *GAPDH*, an example of protein-coding gene, showed a clear accumulation of mNET-seq reads over the termination region, and was substantially shifted downstream following CPSF73 depletion. Whereas the lncRNA metagene pattern, including the example of lncRNA TUG1 profile for T4ph-mNET-seq, also contained some 3′-end signal, depletion of CPSF73 did not affect this profile. This observation suggested that TUG1/lncRNA termination does not rely on CPSF73 activity (118). It has been proposed that the T4ph signal across lncRNA transcription units reflects their less efficient RNA splicing and 3′-end processing, with termination occurring across the entire transcription unit. T4ph appears PAS-dependent also in the context of non-coding transcripts – as transcription profiles are consistent with premature termination at weak PASs in lincRNA genes. However, further studies are necessary to verify whether T4ph is a reliable marker for non-coding transcript termination.

### Timing of T4ph in transcription termination

For several years, the prevailing consensus in the field has been that both allosteric changes in Pol II upon transcribing an active PAS, as well as action of an exonuclease torpedo (XRN2) getting access to the RNA upon PAS cleavage by the endonuclease CPSF73, contribute to transcription termination. Acute depletion of both these nucleases results in extensive termination defects, as shown by the West lab. Experiments performed by Eaton *et al.* show that CPSF73 functions upstream of modifications to the elongation complex and provides an entry site for the XRN2 torpedo (32). These events are underpinned by protein phosphatase 1 (PP1) activity, the inhibition of which extends read-through in the absence of XRN2. These findings support a combined allosteric/torpedo mechanism, in which PP1-dependent slowdown of polymerases over termination regions facilitates their capture by XRN2, following PAS processing. The loss of XRN2 led to clear stabilization of read-through RNA just downstream from the PAS, but this effect dissipated by 20 kb. In the absence of CPSF73, the stabilization of read-through RNA was maintained over 20 kb. This difference is consistent with the idea that polymerases pause over termination regions in the absence of XRN2, but not when CPSF73 is depleted. This pausing is likely due to slow elongation rather than complete arrest, as Pol II can still incorporate 4-thiouridine in the 3′ flanking region even absence of XRN2. By conducting ChIP on two model genes: *MYC* and *ACTB*, the above study also assayed, whether the difference in Pol II behavior caused by XRN2 versus CPSF73 depletion is associated with any changes in T4ph mark. XRN2 loss caused an increase in T4ph signal in the assayed regions immediately downstream from the PAS, similar to the increase in total Pol II occupancy observed in ChIP. However, T4ph was reduced in the same regions when CPSF73 was eliminated. The authors conclude that T4ph is therefore downstream from CPSF73 activity but upstream of XRN2-dependent transcriptional termination (32). Interestingly, there is a slight increase in T4ph ChIP signal upon CPSF73 depletion in the most downstream amplicons assessed in both model genes, similar to increased Pol II ChIP in this region. These findings suggest that T4ph spreads further downstream in the absence of CPSF73. A future genome-wide study could provide more nuanced insights into the role of T4ph in transcription termination.

### Transcription termination in stress conditions – transcripts downstream of genes

In 2015, Joan Steitz’s laboratory published the discovery that stress conditions induce widespread transcription termination defects in human cells. The transcripts emerging beyond typical termination sites were called DoGs (for transcripts extending downstream of genes) (147). Further studies confirmed those findings and provided more detail on the biogenesis of DoGs, as reviewed in (148). In particular, DoGs have been reported to be dependent on Integrator rather than the cleavage and polyadenylation complex (149).

Among various cellular stress conditions that induce termination defects, oxidative stress plays a significant role. Interestingly, in the study conducted earlier by Dirk Eick’s laboratory (77), it was observed that CTD T4 kinase Plk3 can be activated by hypoxia/reoxygenation stress and, therefore, T4, as its target, may fulfill a specialized role in transcription regulation under stress conditions. It was therefore tested whether oxidative stress specifically modulates T4ph levels. Indeed, treatment of HeLa cells with H_2_O_2_ for 30 min upregulated Plk3 protein and T4ph levels in CTD concomitantly, but did not affect the phosphorylation levels of other residues in CTD (77). To uncover the relationship between increased T4ph and stress-induced transcription termination defects, further research is needed.

### Mechanism of Pol II CTD-T4ph function in transcription termination – T4ph as a binding platform in metazoans

Multiple lines of evidence reported here demonstrate an association between Pol II CTD-T4ph and transcription termination. However, correlation does not imply causation, and it is currently unclear whether T4ph is required for transcription termination. The following indirect evidence points in this direction: (i) T4ph occurs specifically in termination regions, (ii) CTD T4 residue is essential for life in metazoan cells, (iii) Pol II CTD is dispensable for initiation and elongation but necessary for transcription termination and (iv) perturbations of T4ph levels – such as oxidative stress in human cells or depletion of phosphatase Dis2 in *S. pombe* – are associated with termination defects.

If T4ph is indeed functionally involved and perhaps even necessary for transcription termination, two plausible and non-exclusive mechanisms are conceivable: it could serve as a platform for the binding of termination factors and/or prevent the binding of elongation factors or anti-terminator proteins to Pol II. Some evidence exists for both of those possibilities.

There is mounting evidence that multiple termination factors in yeast can bind both S2ph and T4ph, summarized in the first part of the review. However, human CTD-phospho-binding studies so far focused on S5 and S2 phosphorylation. A newly published study shows for the first time, that similarly to yeast, many human CID containing proteins can bind T4ph with good affinities (143). It is also interesting to note in this respect, that the majority of proteins with CIDs are termination factors.

On the other hand, a possible mechanism of CTD-T4ph action is preventing the binding of elongation factors. In line with this possibility, an interactome analysis of chromatin-associated Pol II using a modified IP protocol revealed that fewer interacting proteins undergo co-IP with Pol II CTD-T4ph compared to other CTD modification states. In particular, T4ph IP was devoid of splicing and elongation factors (117). Those observations are compatible with T4ph marking pre-termination Pol II. Admittedly, it cannot be ruled out that in this experiment epitope occlusion was a specific issue for T4ph modified CTD/anti-T4ph antibody. Another caveat of chromatin fractionation is the use of 1 M urea, which disrupts weak interactions. No CPA factors were found associated with either S2ph or T4ph CTD in the chromatin fraction *in vivo*, including the CID-containing PCF11 protein, likely due to this reason (117).

A recent elegant study from the Passmore lab has shown that *S. cerevisiae* Pol II can form a stalk-to-stalk dimer, which is compatible with transcription, but not with the binding of transcription elongation factors. The formation of this dimer is promoted upon Pol II dephosphorylation *in vitro* (150). It is proposed that Pol II dimerization reflects an allosteric change required for transcription termination. It would be interesting – although technically challenging – to determine whether Pol II dimers in human cells are modified with T4ph under *in vivo* conditions.

## Conclusions

Currently, there is insufficient evidence to claim that Pol II CTD-T4ph is a modification required for transcription termination. Thus, understanding the functional relationship between the T4ph mark and transcription termination requires further work. At the same time, we argue that the evidence supporting Pol II CTD-T4ph as a reliable genome-wide transcription termination marker is strong. T4ph robustly and specifically marks termination regions at the end of protein-coding genes and correlates well with a decrease in nascent RNA signal. Additionally, CTD-T4ph also allows the detection of premature termination (transcription attenuation) within gene bodies. This is significant, because premature termination is particularly challenging to pinpoint genome-wide in eukaryotes, yet it serves as a potent regulatory mechanism.

The chromatin field has shown that protein post-translational modifications can be powerful markers. Laboratories worldwide use γH2AX as marker for DNA damage, H3K4me3 as a marker for promoters and H3K4me1/H3K27ac as markers for enhancers. For instance, the introduction of H3K4me1/H3K27ac marks has allowed for the convenient mapping of enhancer regions genome-wide and accelerated the study and understanding of enhancers’ influence on gene regulation. Furthermore, this utility is entirely independent of the question of whether these marks are important for enhancer function.

Molecular markers are also frequently used in clinical oncology. While there is often no evidence for the marker being the underlying cause of disease, they can help to accurately classify the patient into a specific group or disease subtype and speed up assigning appropriate treatment. According to the Cambridge dictionary, a marker in biology is defined as ‘a characteristic feature, gene or substance that shows something is happening or likely to happen, or shows which person or animal something comes from’ [‘Marker’ definition (2024) Cambridge Dictionary].

In our view, a wider usage of Pol II CTD-T4ph as a transcription termination marker could impact and benefit (i) more comprehensive genomic annotations, (ii) understanding of regulatory regions and their interplay, (iii) study of mechanisms of transcription and (iv) annotating disease-associated mutations outside of coding regions, and other aspects studied by the nucleic acids research community.

## Data Availability

The code used in this paper can be accessed at https://zenodo.org/records/14237542. The processed data files can also be viewed in public UCSC sessions: T4ph mNET-seq versus ChIP-seq (mNETseq-ChIPseq) and Nascent RNA-seq methods versus T4ph mNET-seq (vs-T4phmNETseq).
